# MicroRNAs Involvement in Radioresistance of Head and Neck Cancer

**DOI:** 10.1155/2017/8245345

**Published:** 2017-02-23

**Authors:** Parwez Ahmad, Jiri Sana, Marek Slavik, Pavel Slampa, Pavel Smilek, Ondrej Slaby

**Affiliations:** ^1^Central European Institute of Technology, Masaryk University, Brno, Czech Republic; ^2^Department of Comprehensive Cancer Care, Masaryk Memorial Cancer Institute, Faculty of Medicine, Masaryk University, Brno, Czech Republic; ^3^Department of Radiation Oncology, Masaryk Memorial Cancer Institute, Brno, Czech Republic; ^4^Department of Otorhinolaryngology and Head and Neck Surgery, St. Anne's Faculty Hospital, Masaryk University, Brno, Czech Republic

## Abstract

Resistance to the ionizing radiation is a current problem in the treatment and clinical management of various cancers including head and neck cancer. There are several biological and molecular mechanisms described to be responsible for resistance of the tumors to radiotherapy. Among them, the main mechanisms include alterations in intracellular pathways involved in DNA damage and repair, apoptosis, proliferation, and angiogenesis. It has been found that regulation of these complex processes is often controlled by microRNAs. MicroRNAs are short endogenous RNA molecules that posttranscriptionally modulate gene expression and their deregulated expression has been observed in many tumors including head and neck cancer. Specific expression patterns of microRNAs have also been shown to predict prognosis and therapeutic response in head and neck cancer. Therefore, microRNAs present promising biomarkers and therapeutic targets that might overcome resistance to radiation and improve prognosis of head and neck cancer patients. In this review, we summarize the current knowledge of the functional role of microRNAs in radioresistance of cancer with special focus on head and neck cancer.

## 1. Introduction

Head and neck cancers are the sixth most common cancers worldwide that are represented mainly by the squamous cell carcinoma (HNSCC) occurring in the oral cavity, pharynx, or larynx. Etiological causes of HNSCC development are excessive use of tobacco and alcohol and infection with human papilloma virus (HPV), especially HPV 16 and HPV 18, and Epstein-Barr virus (EBV) [1, 2]. The current standard therapy consists of radical surgical resection followed by adjuvant radiotherapy (RT) in monotherapy or concomitantly with chemotherapy or targeted therapy. However, the biological behavior of HNSCC is unpredictable at this time and there are many patients resistant to the administrated therapy.

In this regard, great efforts are being made for elucidation of radioresistance mechanisms as well as discovery of new prognostic and predictive biomarkers in response to the ionizing radiation (IR) that remains one of the cornerstones of head and neck cancer treatment [2]. IR may affect cellular components directly and/or indirectly by the generation of highly reactive oxygen species, known as free radicals, that react with other molecules in very short time. The major effect of this process in cells is DNA double-strand breaks (DSB), which ultimately lead to the extensive genome instability and cell death. Nevertheless, there are cellular mechanisms and tumor microenvironment factors that repair and/or prevent these molecular changes, respectively. High activity of these mechanisms supported by tumor microenvironment led to the increased resistance of cancer cells to the IR and, subsequently, to the RT failures, early tumor recurrences, and poor prognoses of cancer patients [3].

Many recent studies have showed that very important molecular players closely related to the response to the IR at the cellular level are among other microRNAs (miRNAs). MiRNAs are endogenous, evolutionary conserved, small noncoding RNAs, 18–25 nucleotides in length, which regulate gene expression by binding to 3′UTR of targeted mRNA. MiRNAs are involved in the regulation of all important cellular processes associated with response to the IR such as DNA damage response and repair, apoptosis, proliferation, and angiogenesis. Moreover, these molecules have been published to be deregulated in many cancers including HNSCC [2, 4] and, thus, miRNAs seem to be key regulators of HNSCC response to the IR. The aim of this review is to summarize present knowledge about radioresistance of HNSCC with emphasis on role of miRNAs in this tumor phenomenon.

## 2. Radioresistance in Head and Neck Cancer

Tumor resistance to the ionizing radiation is a complex process depending on the many biological factors and cellular mechanisms regulated by the intrinsic cell signaling network. The most frequently mentioned events leading to the development of HNSCC radioresistance are disruption of proliferation, apoptosis, DNA damage response and repair, and angiogenic signaling in the tumor [29–31].

### 2.1. Alterations in EGFR, PI3K/AKT, and RAS Pathways

Very important signaling pathway regulating mechanisms mentioned above seem to be EGFR (epidermal growth factor receptor) signaling network triggered canonically by the transmembrane protein with tyrosine kinase activity that is overexpressed in about 90% of HNSCC. High EGFR expression correlates with poor prognosis of patients and resistance to conventional radiotherapy. Most studies have also demonstrated a lower local control after radiation of tumors overexpressing EGFR. The protein overexpression is thought to result from enhanced transcription, whereas gene amplification has been observed less frequently [30, 32]. Stimulation of this receptor may activate many downstream molecules, such as PI3K (phosphatidylinositol-3-kinase) and RAS. PI3K is able to activate AKT that phosphorylates multiple downstream effectors controlling cell survival and apoptosis as well as DNA damage repair and epithelial to mesenchymal transition. In turn, RAS is able to directly stimulate a tyrosine kinase cascade, which comprises in order B-Raf, MEK, and finally mitogen-activated protein kinase (MAPK). MAPK phosphorylates Myc, FOS, and Jun, allowing their nuclear translocation, which leads to cell proliferation [3].

### 2.2. Deregulation of TP53 Associated Intrinsic Apoptosis

TP53 is a tumor suppressor gene which is a key regulator of genome stability through the modulation of DNA damage response. About 40–70% of HNSCC has mutation in TP53 gene, leading to inactivation of its protein product [30, 33]. Alteration in p53 leads to an impaired capability of cell cycle arrest and to inhibition of the apoptosis. As a consequence, tumor cells carrying TP53 mutation are less sensitive to radiation-induced cell death and are unable to restore DNA integrity, thus accumulating several genetic mutations which lead to increased tumor heterogeneity and finally to resistance to conventional radiotherapy [3, 30]. Moreover, several evidences suggest that it is necessary to distinguish carefully TP53 mutations, which are related to the prognosis in HNSCC. In the other words, prognosis of patients varies depending on the particular mutated TP53 protein domain [30, 34, 35]. One of the main classifications of TP53 mutations divide these to disruptive and not disruptive; any mutation in L2 or L3 loop of the DNA binding domain resulting in a polarity change of the protein or any stop codon was classified as disruptive. Disruptive TP53 mutations were associated with poor outcome and increased radioresistance in HNSCC [30, 36, 37].

### 2.3. Hypoxia Induced Neovascularization

Although angiogenesis plays the main role, it is not the only way to develop a vasculature. Another mechanism is called vasculogenesis and is observed mainly in the stage of embryo development where it gives rise to the first primitive vascular plexus from circulating cells. Angiogenesis is then responsible for the remodeling and expansion of this network [38]. Because tumor irradiation abrogates local angiogenesis, the tumor must rely on the vasculogenesis pathway for regrowth after irradiation. Irradiated tumor produces a marked influx of CD11b+ macrophages into the tumor, and these are crucial to the formation of new blood vessels. This process is driven by increased tumor hypoxia that manifests in stabilization of HIF-1*α* and HIF-1*β* heterodimer (hypoxia-inducible factor 1) and upregulation of SDF-1 (stromal cell-derived factor 1 or CXCL12). Disruption of the above signalization prevents the radiation-induced influx of the CD11b+ macrophages and delays tumor recurrence [39–41].

HIF1 also induces proangiogenic growth factors such as FGF (fibroblast growth factor) and VEGF (vascular endothelial growth factors) that stimulate endothelial cells to secrete several proteases and plasminogen activators resulting in the degradation of the vessel basement membrane, which in turn allows cells to invade the surrounding matrix. The cells migrate, proliferate, and eventually differentiate to form a new vessel [42]. Finally, VEGF is also able to activate PI3K/AKT and RAS/MAPK signaling pathways and, thus, is involved in the control of cell survival, proliferation, and apoptosis as described above.

### 2.4. Epithelial-Mesenchymal Transition

EMT (epithelial-mesenchymal transition) is a process that allows a polarized epithelial cell, which normally interacts with basement membrane via its basal surface, to undergo multiple biochemical changes that enable it to assume the mesenchymal cell phenotype, which includes enhanced migratory capacity, invasiveness, elevated resistance to apoptosis, and greatly increased production of ECM components [43, 44]. A crucial step of EMT is the loss of E-cadherin, a strong epithelial marker involved in adherent junction that anchors epithelial cells to each other. Loss or decrease of E-cadherin expression causes the translocation of *β*-catenin protein from the cell membrane to the nucleus to induce transcription of EMT-related genes, such as TWIST and SNAIL1. Another important protein involved in EMT is vimentin, which is an intermediate filament protein used as a marker for mesenchymal cells and is associated with the migratory phenotype, local recurrence, and survival in HNSCC [29, 30]. Emerging evidence suggests that EMT has also a crucial role in cancer radiation resistance primarily via enhancing self-renewal and other CSCs (cancer stem cells) characteristics [45–47].

### 2.5. Cancer Stem Cells

Most cancers, including HNSCC, contain a small subpopulation of cells that, like the stem cells, are characterized by the ability of self-renewal, unlimited and slow proliferation, and differentiation potential. Moreover, these cellular populations called cancer stem cells (CSCs) have also unique potential for tumor initiation and are highly resistant to both chemotherapy and ionizing radiation [48]. Conventional therapeutic approaches are successful in debulking the tumor through targeting of highly proliferating cells. However, as outlined above, the slow-growing CSCs evade conventional therapies, and, with the passage of time, these cells are activated and regenerate tumors locally or at distant sites [49]. This is one of possible causes explaining the relatively high recurrence rates in patients with HNSCC. CSCs are located in the invasive fronts of HNSCC close to blood vessels and express markers such as ALDH (aldehyde dehydrogenase), CD133, CD24, and CD44 [50, 51]. In HNSCC patients, high percentage of CD44 positive cells was associated with higher rate of treatment failure in general, while cells expressing CD44, CD24, Oct4, and integrin *β*1 were associated with poor outcome after radiotherapy [52].

## 3. Involvement of miRNAs in Radioresistance of Cancer

It has been suggested that miRNAs can modulate tumor radioresistance by affecting radiation-related signaling pathways which are involved in DNA damage repair, cell cycle regulation, apoptosis, neovascularization, and inflammation. Among these pathways are EGFR, PI3K/AKT, NF-kB, RAS-MAPK, and TGF-*β*, as well as JAK-STAT signaling [53, 54]. Well described miRNA that regulates response to the ionizing radiation through inhibition of PI3K/AKT pathway is miR-21. Interestingly, this process was also accompanied by the enhancement of autophagy [55]. Abnormal expression of, inter alia, miR-21 has been associated also with radioresistance in lung carcinoma stem-like cells [56] and found in high-risk HPV positive cervical cancer cells where this miR-21 regulates LATS-1 (large tumor suppressor kinase 1) [57]. Glioma radioresistance is via AKT signalization regulated also by the miR-221/222. In this case, activation of AKT has been independent of PTEN status [58]. Nevertheless, the same miRNAs target PTEN and regulate radioresistance in gastric carcinoma cells [59]. PTEN/PI3K/AKT signaling pathway is further regulated by miR-20a. Activation of this pathway results in induction of radioresistance in hepatocellular carcinoma [60]. On the other hand, increased radiosensitivity of hepatic origin tumors has been described after downregulation of miR-210 [61], whereas the same miRNA is also involved in HIF-1*α*/Bcl-2 guided radiosensitivity in colon cancer [62]. The close relationship of miR-210 with hypoxia induced cellular processes has been observed also in cervical and breast cancer [63]. Other miRNAs described in relation to breast carcinoma radioresistance are miR-200c, miR-155, and miR-205. However, the molecular mechanisms of their action are not identical. Whereas miR-200c has been found to enhance radiosensitivity by inhibiting autophagy by targeting UBQLN1 [64], miR-155 regulates DNA repair and enhances radiosensitivity by targeting RAD51 [65]. MiR-205 has been published to be downregulated in radioresistant breast cancer cells and targets ZEB1 and Ubc13 [66]. In glioblastoma, miR-128a, miR-135b, miR-1, miR-125a, miR-150, and miR-425 were found to be associated with radioresistance [67–69]. Moreover, our recent study suggests that miR-338-5p sensitizes glioblastoma cells to radiation through regulation of genes involved in DNA damage response such as NDFIP1 (Nedd4 Family Interacting Protein 1), RHEB (RAS homolog enriched in brain), and PPP2R5A (Protein Phosphatase 2 Regulatory Subunit B′, Alph) [70]. In lung cancer loss of miR-1323 and/or miR-18a enhances radiosensitivity whereas miR-511 has been found to suppress growth of radioresistant cell lines [71–73]. More deep study on molecular mechanisms revealed that effect induced by miR-1323 is mainly through the suppressing of PRKDC (Protein Kinase, DNA-Activated, Catalytic Polypeptide) [71]. Finally, both miR-100 and miR-124 have been found to be associated with radiosensitivity in colorectal carcinoma trough targeting PRRX1 (Paired Related Homeobox 1) [74, 75].

## 4. Involvement of miRNAs in Radioresistance of Head and Neck Cancer

Biological mechanisms and signaling network responsible for radioresistance of HNSCC are very similar to other cancers described above. Therefore, it is not surprising that there are growing evidence supporting the role of miRNAs in response to ionizing radiation also in carcinomas of the head and neck. In HNSCC, ATM-mediated radiosensitivity is regulated by downstream targets such as SMC1 and Snail. In absence of ATM function, miR-16, miR-29b, miR-1254, and miR-150 were found to be upregulated, while let-7e was downregulated [8]. Interestingly, overexpression of miR-150 in glioblastoma promotes radioresistance through upregulation of the cell cycle checkpoint response and its direct targeting led to the higher sensitivity of glioblastoma cells to irradiation [69]. Another study has described that miR-196a promotes an oncogenic effect in HNSCC by suppressing ANXA1 and thus enhancing radioresistance [9]. Similarly, miR-210 is associated with radioresistance in HNSCC, whereas its higher expression has been found in hypoxic cells [10]. Li et al. published a comprehensive study focused on miRNAs in nasopharyngeal carcinoma (NPC). Using next-generation sequencing technology, they revealed miR-371a-5p, miR-34c-5p, and miR-1323 to be overexpressed while miR-324-3p, miR-93-3p, and miR-4501 were downregulated in radioresistant NPC cells [11]. Oncogenic feature of miR-1323 was supported also by Li et al. who published that knockdown of miR-1323 restores sensitivity to radiation in radiation-resistant lung cancer cells [71], whereas miR-324-3p has been found downregulated in radioresistant NPC cells and directly targets WNT2B gene [21]. On the other hand, miR-34a, another member of the tumor suppressive miR-34 family, shows rather opposite effect on radioresistance than described in Li et al.'s study. Hypofractionated radiotherapy induced miR-34a expression and enhanced apoptosis in NPC cells [22]. In these cells, miR-205 downregulation determined radioresistance by targeting of PTEN [12], miR-451 increases radiosensitivity by targeting RAB14 (RAS related protein 14) [5], miR-23a is associated with modulation of radioresistance by targeting IL8 [13], and miR-185-3p regulates radioresistance, similar to miR-324-3p, by targeting WNT2B [23]. Low expression of miR-203 has been found to be correlated with local disease recurrence after radiotherapy in a series of patients with laryngeal cancer [27] whereas, in NPC, miR-203 plays important role in radiosensitivity by targeting IL8/AKT [28]. On the other hand, downregulation of miR-21 enhances radiosensitivity, but the responsible mechanism has to be clarified in NPC. MiR-21 is deeply characterized in ESCC, where its overexpression is related to decrease of PTEN expression in radioresistant cell lines [16, 17]. In ESCC, the sensitivity to the radiation is regulated also by miR-381 and miR-96. Expression levels of miR-381 positively correlate with good response of cells in vitro [24], miR-96 shows opposite effect, and its upregulation led to loss of radiosensitivity [14].

**Figure 1:**
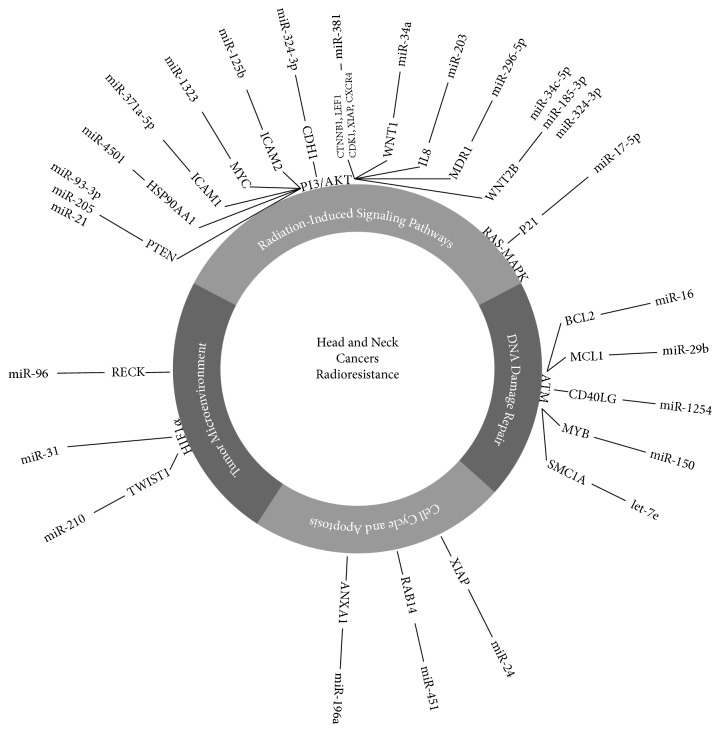
MiRNAs involved in radioresistance of head and neck cancer. This figure shows a regulation of key processes involved in head and neck cancers radioresistance through miRNAs and their targets. ANXA1: Annexin 1, BCL2: B-cell CLL/Lymphoma 2, ATM: Ataxia Telangiectasia Mutated, MCL1: Myeloid cell Leukemia 1, CD40LG: CD40 Ligand, MYB v-Myb: avian myeloblastosis viral oncogene homolog, SMC1A: Structural Maintenance of Chromosomal 1 A, CDH 1: Cadherin 1, PTEN: Phosphatase and Tensin homolog, XIAP: X-linked Inhibitor of Apoptosis E3 ubiquitin Protein, WNT2B: wingless-type MMTV integration site family member 2B, RAB14: member of RAS oncogene family, HIF-1*α*: hypoxia-inducible factor 1 alpha subunit, TGF-WNT: transforming growth factor-wingless-type MMTV integration site family, RECK: reversion-inducing cysteine rich protein with Kazal motifs, MDR1: Multi-Drug Resistance gene 1, IL8: Interleukin 8, HSP90AA1: Heat Shock protein 90 kDa Alpha (cytosolic) Class A Member 1, ICAM 1: Intercellular Adhesion Molecule 1, Myc v-Myc: Avian Myelocytomatosis viral oncogene homolog, ICAM 2: Intercellular Adhesion Molecule 2, CTNNB1: Catenin (Cadherin associated protein) Beta1, LEF1: Lymphoid Enhancer-Binding Factor 1, CDK1: Cyclin-Dependent Kinase 1, CXCR4: Chemokine (c-x-c motif) Receptor 4, WNT1: wingless-type MMTV integration site family member 1, TWIST1: Twist Basic Helix-Loop-Helix Transcription Factor 1.

In LSCC (laryngeal squamous cell carcinoma), upregulation of miR-296-5p regulates radiosensitivity by targeting MDR1 gene [15]. Upregulation of miR-24 has been found to be associated with radiosensitivity through targeting of XIAP (X-linked inhibitor of apoptosis protein) in LSCC and NPC [19, 20]. In OSCC (oral squamous cell carcinoma), miR-125b regulates proliferation and radiosensitivity by targeting ICAM2 [25] and miR-17-5p regulates expression of p21 [26]. At the end, upregulation of miR-31 enhances radiosensitivity in esophageal adenocarcinoma cell lines probably by activating HIF1 [6, 7]. An involvement of miRNAs in the radioresistance of head and neck cancer is summarized in Table 1 and Figure 1.

## 5. Conclusion

Head and neck cancers account for about 3% of all cancers in developed countries and their etiology is associated with tobacco, alcohol, and sexually transmitted infection of human papilloma virus and Epstein-Barr virus. Therefore, it is possible to assume that the incidence will rise in the future. The biological behavior and prognosis of head and neck cancer is unpredictable at this time, radiotherapy often fails, and early recurrences are observed. This is primarily caused by the resistance of tumors to radiotherapy. Overcoming the resistance is nowadays one of the main challenges in head and neck cancers research. Recently, several signaling pathways have been described to be associated with resistance of head and neck cancer cells to ionizing radiation. Many genes involved in these pathways are regulated by miRNAs, and direct deregulation of many miRNAs was shown to affect sensitivity to radiation in cellular models. Thus, miRNAs are promising both prognostic and predictive markers, as well as therapeutic targets, which may enable overcoming the resistance of head and neck cancers to the radiotherapy and improve therapeutic results in patients afflicted with these malignancies.

## Figures and Tables

**Table 1:** Expression of radioresistance associated miRNAs in head and neck cancers.

Expression in tumor	miRNA	Carcinoma type	Reference
Upregulated	miR-451	NPC	[5]
	miR-31	OAC	[6, 7]
	miR-150	HNSCC	[8]
	miR-1254	HNSCC	[8]
	miR-16	HNSCC	[8]
	miR-29b	HNSCC	[8]
	miR-196a	HNSCC	[9]
	miR-210	HNSCC	[10]
	miR-1323	NPC	[11]
	miR-34c-5p	NPC	[11]
	miR-371a-5p	NPC	[11]
	miR-205	NPC	[12]
	miR-23a	NPC	[13]
	miR-96	ESCC	[14]
	miR-296-5p	LSCC	[15]
	miR-21	NPC	[16, 17]

Downregulated	miR-324-3p	NPC	[11]
	miR-141	ESCC	[18]
	miR-18b	ESCC	[18]
	miR-301a	ESCC	[18]
	miR-24	LSCC, NPC	[19, 20]
	miR-let 7e	HNSCC	[8]
	miR-4501	NPC	[11]
	miR-93-3p	NPC	[11]
	miR-324-3p	NPC	[21]
	miR-34a	NPC	[22]
	miR-185-3p	NPC	[23]
	miR-381	ESCC	[24]
	miR-125b	OSCC	[25]
	miR-17-5p	OSCC	[26]
	miR-203	LSCC, NPC	[27, 28]

HNSCC: head and neck squamous cell carcinoma; NPC: nasopharyngeal carcinoma; OSCC: oral squamous cell carcinoma; OAC: oral adenocarcinoma; LSCC: laryngeal squamous cell carcinoma; ESCC: esophageal squamous cell carcinoma.
